# Neural Adaptation Effects in Conceptual Processing

**DOI:** 10.3390/bs5030353

**Published:** 2015-07-31

**Authors:** Barbara F. M. Marino, Anna M. Borghi, Luca Gemmi, Cristina Cacciari, Lucia Riggio

**Affiliations:** 1 Dipartimento di Psicologia, Università di Milano-Bicocca, 1, P.zza dell’Ateneo Nuovo, 20125 Milano, Italy; 2 Dipartimento di Neuroscienze, Università di Parma, 39/E, Via Volturno, 43125 Parma, Italy; E-Mails: luca.gemmi@gmail.com (L.G.); lucia.riggio@unipr.it (L.R.); 3 Dipartimento di Psicologia, Università di Bologna, 5, Viale Berti Pichat, 40127 Bologna, Italy; E-Mail: anna.borghi@gmail.com; 4 Istituto di Scienze e Tecnologie della Cognizione, CNR, 44, Via S. Martino della Battaglia, 00185 Roma, Italy; 5 Dipartimento di Scienze Biomediche, Metaboliche e Neuroscienze, Università di Modena, 287, Via Giuseppe Campi, 41125 Modena, Italy; E-Mail: cristina.cacciari@unimore.it

**Keywords:** grounded cognition, conceptual processing, neural adaptation, McCollough effect

## Abstract

We investigated the conceptual processing of nouns referring to objects characterized by a highly typical color and orientation. We used a go/no-go task in which we asked participants to categorize each noun as referring or not to natural entities (e.g., animals) after a selective adaptation of color-edge neurons in the posterior LV4 region of the visual cortex was induced by means of a McCollough effect procedure. This manipulation affected categorization: the green-vertical adaptation led to slower responses than the green-horizontal adaptation, regardless of the specific color and orientation of the to-be-categorized noun. This result suggests that the conceptual processing of natural entities may entail the activation of modality-specific neural channels with weights proportional to the reliability of the signals produced by these channels during actual perception. This finding is discussed with reference to the debate about the grounded cognition view.

## 1. Introduction

In the last decades, an increasing number of functional neuroimaging, neurophysiologic, and behavioral studies have provided support for the grounded cognition view whose main theoretical assumption is that conceptual processing makes use of the same neural systems that mediate perception and action (for empirical reviews and theoretical discussions see [1,2,3,4,5,6,7,8,9,10]). Evidence in support of grounded cognition showed that the comprehension of action-related words and sentences is associated to a fast and somatotopic activation in motor and pre-motor cortices (e.g., [11,12,13]). Research has also shown a modality-specific recruitment of sensory-neural subsystems in comprehension and conceptual retrieval of linguistic materials. For example, activation of the primary olfactory system (*i.e.*, the piriform cortex) was found during the presentation of odor-related nouns (e.g., “cinnamon”; [14]), and activation of the visual color system (*i.e.*, the V4 complex in the left fusiform gyrus) was observed during the presentation of color-related nouns (e.g., “banana”; [15]), relative to modality-neutral words. The engagement of modality-specific neural areas while processing perception-related concepts is also supported by behavioral studies indicating that perceptual phenomena also arise in conceptual retrieval. For example, verifying the properties of concepts incurs in costs, similarly to what happens during perceptual processing when switching between one perceptual modality to another (e.g., [16]).

However, a critical consideration of grounded cognition has recently begun to gain credit (e.g., [6,7,17,18]). The most controversial issue concerns the dynamic of the activation flow between perceptual and motor systems and the conceptual system. Specifically, it has been claimed that the recruitment of the neural areas underlying perception and action may occur subsequently rather than being time-locked to conceptual processing [7]. Hence, activation of the perceptual and motor systems by linguistic material may reflect downstream activation which cascades from an amodal (*i.e.*, symbolic) conceptual system.

Overcoming the objection of downstream activation has proved to be a rather complex and still unresolved issue. It is likely that no single study indeed can by itself resolve such an intricate set of problems. Notwithstanding, one possibility for contributing to this complex debate is to investigate the conceptual processing of objects with highly typical perceptual features when it occurs after a selective suppression or impairment of the sensory areas sensitive to those features. Indeed, finding an impaired performance may be taken to imply an impaired state of the perceptual system that could not be properly activated. Behavioral studies devoted to test this possibility can be designed by taking advantage of neural adaptation to induce a selective deactivation of specialized populations of neurons within the visual cortex. Neural adaptation is a biological process whereby sensory neuron responsiveness decreases over time as a result of persistent stimulation. Neural adaptation is inherently selective to the stimulation characteristics and typically has a short-lasting persistence (e.g., [19]). Besides short-term neural adaptation, long-term neural adaptation can also be induced as shown, for instance, by the adaptation of color-edge sensitive neurons in the posterior LV4 region of visual cortex [20] that lasts hours if not days [21]. This kind of neural adaptation, induced by viewing for a few minutes two alternating orthogonally oriented black gratings (e.g., vertical and horizontal) on backgrounds of opposite chromatic polarity (e.g., red and green), is thought to be responsible of the McCollough effect in which black-and-white orthogonally oriented gratings are perceived as tinged complementarily with the color of the induction backgrounds [22].

The long lasting neural adaptation associated to the McCollough effect is jointly related to both color and orientation [22,23]. In this exploratory study, we adopted the procedure leading to this effect to investigate conceptual processing when a prior selective impairment of the visual system occurred. Specifically, we compared the performance of participants adapted to either black-and-green horizontal gratings or black-and-green vertical gratings. Participants performed a go/no-go categorization task on nouns referring to green and non-green objects commonly perceived as having vertical, horizontal, or squared dimensions (see [24,25] for preliminary investigations of the effects of adaptation to colors and orientations on conceptual decision tasks).

We predicted that if the activation of the visual system is critical for conceptual processing, the categorization of nouns referring to green-vertical objects should be interfered by adaptation to black-and-green vertical gratings. In contrast, the categorization of nouns referring to green-horizontal objects should be interfered by adaptation to black-and-green horizontal gratings. Indeed, these two kinds of neural adaptation selectively decrease the responsiveness of neurons jointly sensitive to the green color and the vertical or horizontal dimension. In addition, we predicted that the adaptation to black-and-green vertical or horizontal gratings should exert no effect on the categorization of nouns that do not refer to objects characterized by the adapted color and dimension (*i.e.*, green-squared objects, non-green vertical objects, non-green horizontal objects and non-green squared objects) since the responsiveness of neurons selectively sensitive to that color or dimension should remain the same.

## 2. Materials and Methods

### 2.1. Participants

Thirty-four students of the University of Parma (20 females, mean age: 24.1 years, SD:3.5) took part in the experiment as unpaid volunteers. Sample size was calculated *a priori* using GPower software (version 3.1, Universität Kiel, Kiel, Germany) to obtain a statistical power of 0.90 (Cohen’s effect size for F test = 0.10) with an α error probability of 0.05 in the categorization task (see below). All participants were right-handed native Italian speakers and had normal color vision, as confirmed using Ishihara’s plates [26]. None of them had any prior knowledge of the McCollough effect. Participants were unaware of the purpose of the experiment and gave their informed consent before testing. The study was conducted in accordance with the ethical standards laid down in the 1964 Declaration of Helsinki and fulfilled the ethical standard procedure recommended by the Italian Association of Psychology (AIP). All the experimental protocols were also approved by the Ethics Commission of Parma University.

### 2.2. Procedure

Participants were tested individually in a sound-attenuated room, dimly illuminated by a halogen lamp directed towards the ceiling. They sat comfortably in front of the screen of a computer monitor (a Philips 14 inch color CRT monitor with a 1024 × 768 pixel resolution, interfaced with an Pentium 2.80 GHz computer equipped with a NVIDIA GeForce 7300 LE Video Board) with their head supported by a chin rest in order to maintain a stable eye-to-screen distance of 57 cm. The experiment consisted of five phases which were completed in fixed order over one session lasting about 40 min. Throughout the experiment, stimulus presentation and response collection were controlled using the computer software E-Prime, version 1.1. (Psychology Software Tools, Inc., [27]).

#### 2.2.1. Phase 1: Induction of the McCollough Effect

**Figure 1:**
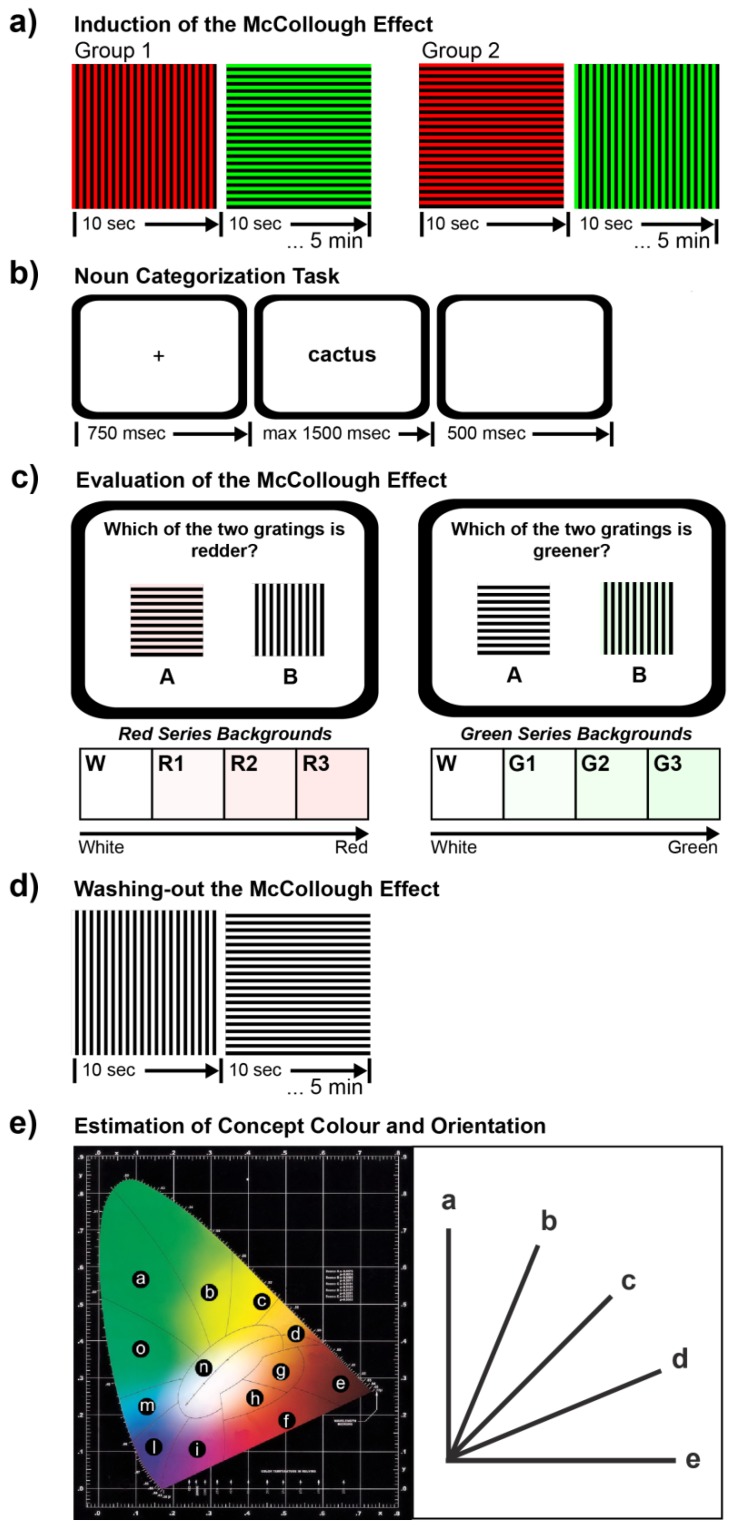
Experimental procedure and materials. (**a**) Orthogonally oriented grating patterns used for each group of subjects to induce a McCollough effect of inverse polarity, and timing of stimulus presentation; (**b**) Schematic diagram of trial structure for the noun categorization task; (**c**) Sample of grating pairs for comparisons and color palettes for backgrounds of the red and the green series; (**d**) Achromatic oriented grating patterns used for deleting the McCollough effect; (**e**) Left: the CIE (Comission Internationale de l'Éclairage) color space chromaticity diagram used for the estimation of concept color and its division into regions of different color. Right: diagram used for the estimation of concept orientation.

To decrease the responsiveness of visual neurons in posterior LV4 region, jointly sensitive to the green color and the vertical or horizontal orientation, participants were exposed to a McCollough effect induction procedure in which two orthogonally oriented grating patterns (spatial frequency: 0.75 cycles/degree) were displayed alternatively on the computer screen every 10 s for a total of 5 min. The gratings were squared and subtended 26.66° of visual angle. Participants were randomly assigned to one of two groups. Participants in Group 1 (11 females and 6 males) were presented with red-and-black vertical gratings (Red = (255,0,0) RGB coordinates; Black = (0,0,0) RGB coordinates) and green-and-black horizontal gratings (Green = (0,255,0) RGB coordinates). Participants in Group 2 (9 females and 8 males) were exposed to orthogonally oriented gratings of inverse chromatic polarity as that used in Group 1 (Figure 1a). Participants were instructed to continue viewing the gratings while fixating the center of the screen throughout the induction procedure.

#### 2.2.2. Phase 2: Noun Categorization Task

**Figure 2:**
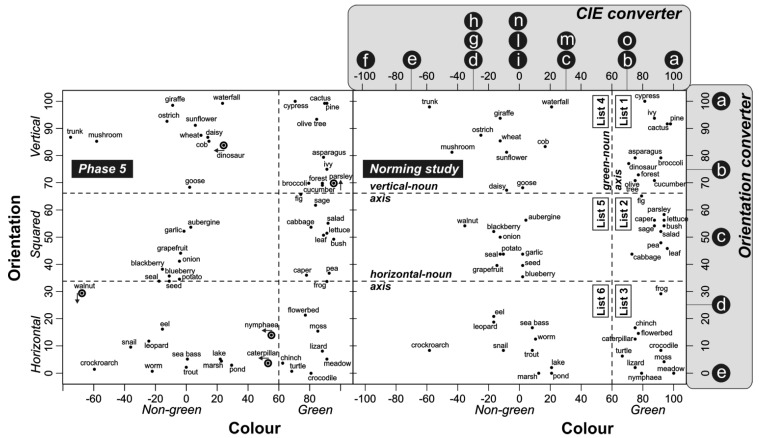
Referents of critical nouns used in the categorization task are represented in a two-dimensional space spanned by estimated color and orientation axes. Location of noun referents in the space is defined by mean values of their color and orientation estimates as measured in Phase 5 (**left** plot) and in the norming study (**right** plot). Tables used for numeric conversion of collected color and orientation estimates are displayed in the upper right corner of the figure. In each plot, two horizontal dashed lines indicate the borders among vertical, squared, and horizontal orientations, whereas a vertical dashed line denotes the border between green and non-green colors. In the right plot (norming study), the intersecting dashed lines subdivide the space into six regions, each containing 10 nouns that refer to objects with the same estimated color and orientation (thus included in the same stimulus list). In the left plot (Phase 5), an arrow marks the nouns that changed their spatial location relative to the norming study (the arrowhead points away from the list originally containing the nouns).

To investigate whether the neural adaptation induced in Phase 1 had an effect on the conceptual processing of objects having both, one or none of the inducing colors and orientations, we used a go/no-go noun categorization task. Stimuli consisted of 120 Italian nouns: 60 nouns referred to natural objects (henceforth critical stimuli; e.g., “cactus”, “turtle”) and 60 referred to man-made objects (e.g., “bell”, “lock”). The critical stimuli were divided into 6 lists, each of which contained 10 nouns (see Table 1). The nouns in the 6 lists referred to green vertical, green squared (*i.e.*, neither vertical nor horizontal), green horizontal, non-green vertical, non-green squared, and non-green horizontal natural objects (e.g., “cactus”, “pea”, “turtle”, “mushroom”, “potato”, and “snail”), respectively. The critical nouns were selected in a norming study on the basis of the color and orientation estimations provided by 12 students not participating in the main experiment (Figure 2). In the norming study, we used the same procedure adopted in Phase 5 of the present study (see below). The nouns in the six lists were matched for word length (6.7, 6.9, 6.8, 6.8, 5.9, and 6.5 letters for nouns referring to green vertical, green squared, green horizontal, non-green vertical, non-green squared, and non-green horizontal natural objects, respectively; *F*(5,54) = 0.35, *p* = 0.88), and lexical frequency (2.23, 1.97, 1.68, 1.79, 3.22, and 2.32 in occurrences per million [28] – ~3,798,000 words; *F*(5,54) = 0.19, *p* = 0.96).

Participants were presented with a blank screen for 500 ms, followed by a fixation point for 750 ms, and then by a noun that was displayed centrally in black lowercase Courier New bold font (size = 24 points). The noun remained visible until participant’s response or until 1500 ms had passed (Figure 1b). The task was to determine, as fast and accurately as possible, whether the noun referred to a natural entity by pressing with the right index finger a computer key centered on their body midline with the right index finger. Participants had to refrain from responding when the noun referred to a man-made object. They received acoustic feedback when they made a response error. The trial order was randomized.

#### 2.2.3. Phase 3: Evaluation of the McCollough Effect

To test whether the adaptation procedure administered in Phase 1 was successful, the McCollough effect was evaluated by means of a visual paired comparison task. Participants were presented with pairs of orthogonally oriented grating patterns (spatial frequency: 0.75 cycles/degree), displayed side by side on the computer screen (Figure 1c). Two series of 28 pairs each were used. The first series (*i.e.*, the red series) was obtained by combining eight different squared grating patterns, each subtending 13.33° of visual angle. Four patterns were black horizontal gratings on white (White = (255,255,255) RGB coordinates) and three light red (Red1 = (255,248,248), Red2 = (255,240,240), and Red3 = (255,233,233) RGB coordinates) backgrounds. The other four patterns were black vertical gratings on the same backgrounds. The second series (*i.e.*, the green series) was the same as the first one, with the exception that three light green (Green1 = (248,255,248), Green2 = (240,255,240), and Green3 = (233,255,233) RGB coordinates) backgrounds were used instead of the light red ones. Each pair of gratings from each series was presented twice (for a total of 112 trials), the second time exchanging the spatial position of gratings.

In each trial, a pair of gratings was centrally displayed on the computer screen. For pairs belonging to the red series, participants were asked to indicate which grating was perceived as redder by pressing the key indicated under the selected grating (method of pair comparisons). For pairs belonging to the green series, participants indicated which grating was perceived as greener. Participants were given as much time as necessary to complete the comparison between gratings and to respond. Trial order was randomized.

If the induction procedure used in Phase 1 was successful, participants adapted to green-and-black horizontal gratings (Group 1) should judge white gratings of the same orientation as redder than white gratings of opposite orientation, and vice versa for participants adapted to green-and-black vertical gratings (Group 2).

#### 2.2.4. Phase 4: Washing-Out the McCollough Effect

To delete the long-term neural adaptation generated at the beginning of the experimental session, the same procedure employed in Phase 1 (Figure 1d) was used but this time participants of both groups were exposed to white-and-black vertical and horizontal grating patterns.

#### 2.2.5. Phase 5: Estimation of Concept Color and Orientation

To further control the critical nouns used in Phase 2 as go-stimuli, participants were asked to judge the color and prevalent orientation of the natural objects denoted by these nouns. For the color estimation, each trial started with a CIE color space chromaticity diagram (Figure 1e, left panel), displayed centrally on the computer screen, and a critical noun written below the diagram. Participants were asked to press the key that identified the region on the CIE diagram containing the color of the noun referent. Immediately after the color estimation response, the CIE diagram was replaced by an image depicting five lines with an orientation ranging from 0 to 90 degrees (Figure 1e, right panel). For the orientation estimation, participants were asked to press the key containing the line that matched the orientation that predominantly characterizes the natural object denoted by the noun.

## 3. Results

### 3.1. Estimation of Concept Color and Orientation

The color and orientation estimates produced for each critical noun were converted into numeric values and averaged across subjects (for the conversion tables used, see Figure 2). The nouns were then projected into a color × orientation space. This space was subdivided into six regions by three intersecting reference lines, namely the green-noun axis, the horizontal-noun axis, and the vertical-noun axis. These axes were identified in the norming study and were employed to assign the nouns to the stimulus lists used in Phase 2. The noun positions obtained in Phase 5 (Figure 2, left plot) were visually compared to those obtained in the norming study (Figure 2, right plot). The comparison showed a good overlapping of the noun positions. Few exceptions occurred. Specifically, the noun “dinosaur” clearly migrated from the green vertical region (list 1) to the center of the non-green vertical region (list 4). Since this item no more clearly fitted the criteria for being reliably included in list 1, it was excluded from the analysis. Migrations of minor entity (*i.e.*, falling very near to the edge of the original lists) were observed for other four nouns (*i.e.*, “parsley”, “walnut”, “nymphaea”, and “caterpillar”; see Figure 2). These items were not removed from the analysis since the criteria for list inclusion were not clear-cut or absolute but were arbitrarily determined on the basis of the color and dimension estimates produced in the norming study, thus allowing some tolerance.

### 3.2. Noun Categorization Task

None of the participants was excluded from the analysis since they were accurate on at least of 90% of the trials. Some participants spontaneously reported difficulties in responding to the noun “cimice” (“chinch”) as it referred to both a natural entity (*i.e.*, a green bug) and a man-made object (*i.e.*, a covert listening device). This ambiguous item was thus excluded from the analysis. Go-trials with missing responses were removed without replacement (0.67% of total go-trials). Since the error rate was extremely low (<5%), errors were not further analyzed. Reaction Times (RTs) below 130 ms (*i.e.*, response anticipations, 0.0% of total go-trials) or above 1000 ms (*i.e.*, time out responses, 2.4% of total go-trials) were excluded from the analysis. These cutoffs corresponded to those used in previous single-word semantic decision tasks (e.g., [12]). Mixed-effects models [29,30] were employed as statistical tool. The effects of interest were those associated to the experimental manipulations—that is, Neural Adaptation (Group 1: green-and-black horizontal gratings *vs.* Group 2: green-and-black vertical gratings), Noun Type (List 1: green vertical *vs.* List 2: green squared *vs.* List 3: green horizontal *vs.* List 4: non-green vertical *vs.* List 5: non-green squared *vs.* List 6: non-green horizontal), and their mutual interaction. In order to account for more error variance, a number of covariates were also considered including lexical variables (*i.e.*, word length and log-transformed written lexical frequency) and semantic variables such as word imageability (*i.e.*, how easily the word evokes a mental image of its referent), familiarity (*i.e.*, how often one encounters the word referent in natural environments), and animacy (*i.e.*, how much the word referent is capable of having self-produced motion and being a causal-agent). Imageability, familiarity and animacy were rated by 12 students not involved in the experiment (8 females and 4 males, mean age: 25.1 ± 3.3 years) using a seven-point scale (0: absent; 6 = extremely present, see Table 1). Lexical and semantic covariates were tested for collinearity by calculating correlations prior to their inclusion in the mixed model. Word imageability and animacy entered into strong correlations with word familiarity (*r* > 0.47, *p* < 0.001; *r* > −0.54, *p* < 0.001, respectively). We therefore de-correlated word imageability from familiarity by regressing imageability on familiarity and taking the residuals as new, orthogonalized, covariate. The same procedure was used for animacy. These residualized variables correlated well with the original measures (*r* = 0.88, *p* < 0.001 for imageability and *r* = 0.84, *p* < 0.001 for animacy) and thus de-correlation did not change the nature of the original measures.

Random intercepts for subjects and items were introduced in the initial model. The fixed factors were Neural Adaptation and Noun Type (with interaction). A forward selection procedure was used to evaluate the effect of by-subjects random slopes for Noun Type, as well as the effects of the covariates. Effects were added only if they significantly improved the model fit, as indicated by likelihood ratio tests. After having identified the best model with the forward selection procedure, atypical outliers were identified and removed (employing 2.5 SDs of the residual errors as a criterion). Statistics in the refitted models are reported. The statistical significance of the fixed parameters was evaluated using the Satterthwaite’s methods for estimating degrees of freedom. The statistical analyses were performed using the R package lmerTest (version 2.0.11., [31]) within the R environment for statistical computing (version 3.1.1., [32]).

If neural adaptation has an effect on conceptual processing, as we hypothesized, we should find longer categorization times for nouns referring to green horizontal entities (stimulus list 3) in Group 1 (where participants were adapted to green-and-black horizontal gratings) than in Group 2 (where participants were adapted to green-and-black vertical gratings). In contrast, longer categorization times should be necessary for green-vertical nouns (stimulus list 1) in Group 2 than in Group 1. Categorizing nouns referring to green squared objects, non-green vertical objects, non-green squared objects, and non-green squared objects (*i.e.*, stimulus lists 2, 4, 5, and 6) should require similar response times in both groups since these nouns do not refer to objects having the adapted color and dimension.

**Figure 3:**
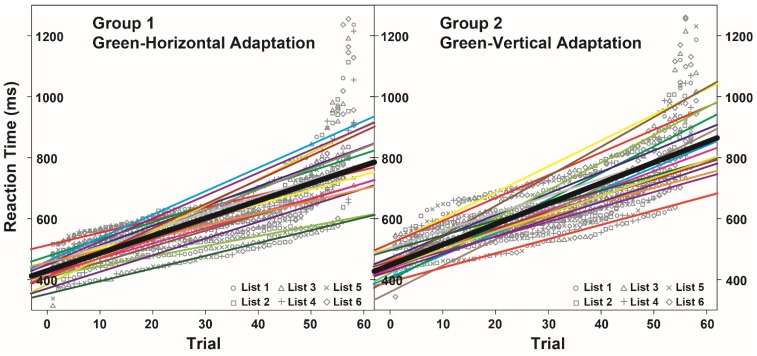
Regression lines (bold black lines) showing the effect of adaptation induced in participants of Group 1 (**left** panel) and Group 2 (**right** panel) on reaction times. Colored thick lines show the effect also for each participant.

In contrast to our predictions, the significant main effect of Neural Adaptation (*F* = 5.33, *p* = 0.028, Figure 3) indicated that noun categorization was about 48 ms slower for participants exposed to green-and-black vertical gratings during the McCollough effect induction procedure than for participants exposed to green-and-black horizontal gratings. Neither the main effect of Noun Type (*F* = 1.36, *p* = 0.256) nor the expected interaction between Noun Type and Neural Adaptation (*F* = 1.78, *p* = 0.113) were significant. Table 2 reports the parameters of the significant effects included in the final model.

The adaptation of neurons jointly sensitive to green-and-black vertical gratings led to an overall slow-down of the conceptual processing of natural entities. Hence, this effect was not restricted to the nouns referring to green vertical objects, as we instead expected.

**Table 1:** Complete list of nouns used in Phase 2, their length (number of letters), lexical frequency (occurrences per million), familiarity (score on a seven-point scale), imageability (score on a seven-point scale), and animacy (score on a seven-point scale).

Italian Noun	English Noun	List	Word Length	Lexical Frequency	Familiarity	Imageability	Animacy
Abete	pine	1	5	0.85	5.25	5.75	2.58
asparago	asparagus	1	8	0.01	5.33	5.83	1.00
Bosco	forest	1	5	17.8	4.83	5.25	2.08
broccolo	broccoli	1	8	0.01	5.17	5.25	1.17
Cactus	cactus	1	6	1.11	4.58	5.67	2.17
cetriolo	cucumber	1	8	0.28	5.50	5.67	1.17
cipresso	cypress	1	8	0.01	5.00	5.42	1.58
dinosauro	dinosaur	1	9	0.68	2.00	5.50	5.00
Edera	ivy	1	5	1.48	5.17	5.58	2.50
Ulivo	olive (tree)	1	5	0.09	5.25	5.67	1.83
cappero	caper	2	7	0.01	5.25	5.50	1.17
cespuglio	bush	2	9	1.47	5.17	5.50	1.92
Fico	fig	2	4	1.15	5.67	5.75	1.17
Foglia	leaf	2	6	6.79	5.75	5.67	1.83
insalata	salad	2	8	4.19	5.75	5.83	1.42
lattuga	lettuce	2	7	0.47	5.50	5.58	1.33
Pisello	pea	2	7	0.18	5.83	5.75	1.58
prezzemolo	parsley	2	10	3.08	5.50	5.75	1.92
Salvia	sage	2	6	2.26	5.67	5.83	1.83
Verza	cabbage	2	5	0.10	5.33	5.25	2.17
Aiuola	flowerbed	3	6	0.02	5.33	5.67	1.08
Bruco	caterpillar	3	5	0.03	4.75	5.50	4.33
cimice	chinch	3	6	0.12	4.83	5.08	5.17
coccodrillo	crocodile	3	11	1.50	3.42	5.75	5.08
lucertola	lizard	3	9	0.01	5.42	5.75	5.50
muschio	Moss	3	7	0.91	4.50	5.25	2.83
Ninfea	nymphaea	3	6	0.01	3.25	5.50	2.08
Prato	meadow	3	5	12.22	5.83	5.83	2.25
Rana	frog	3	4	1.13	4.67	5.67	5.17
tartaruga	turtle	3	9	0.83	4.83	5.67	5.00
cascata	waterfall	4	7	1.85	4.33	5.75	2.58
Fungo	mushroom	4	5	0.59	5.25	5.75	2.50
giraffa	giraffe	4	7	0.01	3.92	5.75	5.00
girasole	sunflower	4	8	0.01	5.17	5.83	2.67
Grano	wheat	4	5	4.95	4.58	5.58	1.17
margherita	daisy	4	10	0.01	5.58	5.83	1.67
Oca	goose	4	3	4.94	4.58	5.67	5.17
pannocchia	corncob	4	10	0.01	5.17	5.67	1.58
struzzo	ostrich	4	7	1.07	3.83	5.67	5.42
Tronco	trunk	4	6	4.51	5.25	5.67	2.08
Aglio	garlic	5	5	3.69	5.58	5.75	0.75
cipolla	onion	5	7	4.10	5.75	5.67	1.08
Foca	seal	5	4	0.25	3.33	5.50	4.83
melanzana	aubergine	5	9	0.12	5.50	5.67	1.50
mirtillo	blueberry	5	8	0.37	5.58	5.83	1.83
Mora	blackberry	5	4	1.10	5.42	5.50	1.67
Noce	walnut	5	4	2.84	5.83	5.83	1.33
Patata	potato	5	6	13.32	5.67	5.67	1.83
pompelmo	grapefruit	5	8	0.03	5.08	5.67	1.17
Seme	seed	5	4	6.39	5.58	5.67	1.67
branzino	(sea) bass	6	8	0.02	3.92	4.25	4.00
Lago	lake	6	4	16.26	5.50	5.83	2.17
leopardo	leopard	6	8	1.13	3.25	5.58	5.50
lumaca	snail	6	6	0.13	5.33	5.58	4.83
murena	eel	6	6	0.01	2.92	5.33	4.67
palude	marsh	6	6	1.33	2.42	5.00	2.00
scarafaggio	cockroach	6	11	0.12	4.42	5.33	5.33
Stagno	pond	6	6	2.30	4.25	5.50	2.08
Trota	trout	6	5	0.66	5.00	5.67	5.33
Verme	worm	6	5	1.26	5.33	5.50	5.17

**Table 2:** Fixed effect of the final mixed model on noun categorization times.

Fixed Effect	Estimate	SE	Df	*t* Value	*P*
Intercept	625.981	44.180	54.6	14.169	0.0001 *
Adaptation: Group2	48.381	19.826	53.5	2.440	0.018 *
Noun Type: 2	27.814	15.710	72.0	1.770	0.081
Noun Type: 3	31.857	16.857	67.8	1.890	0.063
Noun Type: 4	26.765	16.072	70.6	1.665	0.101
Noun Type: 5	6.694	15.574	72.3	0.430	0.669
Noun Type: 6	33.671	17.685	64.6	1.904	0.061
Adaptation: Group2 *					
Noun Type: 2	−24.230	14.309	1753.1	−1.693	0.091
Adaptation: Group 2 *					
Noun Type: 3	−25.708	14.639	1752.1	−1.756	0.079
Adaptation: Group 2 *					
Noun Type: 4	−2.851	14.241	1752.4	−0.200	0.841
Adaptation: Group 2 *					
Noun Type: 5	−3.105	14.242	1752.1	−0.218	0.827
Adaptation: Group 2 *					
Noun Type: 6	6.968	14.410	1752.1	0.484	0.629
Word Animacy	−9.810	3.496	45.9	−2.806	0.007 *
Word Imageability	−67.318	19.940	52.7	−3.376	0.001 *
Word Familiarity	−16.266	6.210	46.3	−2.619	0.012 *
Word Length	5.534	2.221	46.2	2.492	0.016 *
Lexical Frequency	−1.557	1.526	46.2	−1.020	0.313

The reported covariates significantly improved the model goodness of fit. All of them, with the exception of Lexical Frequency, had also significant effects.

### 3.3. Evaluation of the McCollough Effect

**Figure 4:**
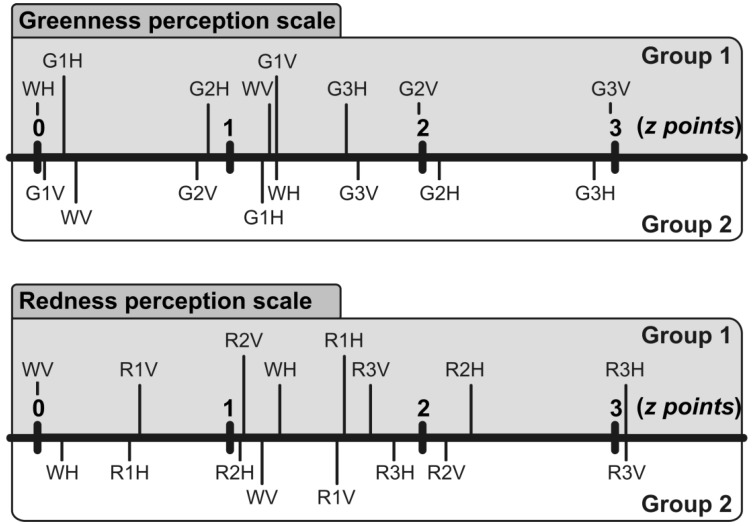
Positions (z points) of grating patterns from the red and the green series on the perceptual scale of both greenness (upper plot) and redness (lower plot), separately for each neural adaptation (Gray field: Group 1—Green-and-black horizontal gratings; White field: Group 2—Green-and-black vertical gratings). Each pattern is identified by a label indicating its background color (e.g., W stands for White, G1 for Green 1, R3 for Red 3, and so on) and grating orientation (e.g., H stands for Horizontal and V for Vertical).

The pair-comparison data collected in Phase 3 were analyzed according to Thurstone’s Case V procedure [33], separately for each group of participants (Group 1 and 2). Two separate scales were constructed (Figure 4), one from pair-comparisons between grating patterns of the green series (*i.e.*, the greenness perception scale) and the other from pair-comparisons between grating patterns of the red series (*i.e.*, the redness perception scale). A visual inspection of Figure 4 reveals that the induction procedure used at the beginning of the experimental session (Phase 1) caused a McCollough effect that lasted beyond the noun categorization task (Phase 2) with comparable strength, but opposite polarity, in the two groups of participants. Indeed, participants adapted to green-and-black horizontal gratings (Group 1) perceived the black horizontal grating on a white background (*i.e.*, WH) as being tinged with light red and the black vertical grating on a white background (*i.e.*, WV) as being tinged with light green. In contrast, participants adapted to green-and-black vertical gratings (Group 2) perceived the black horizontal grating on a white background as being tinged with light green and the black vertical grating on a white background as being tinged with light red. Therefore, we can exclude that our results may reflect a failure in inducing the McCollough effect.

**Figure 5:**
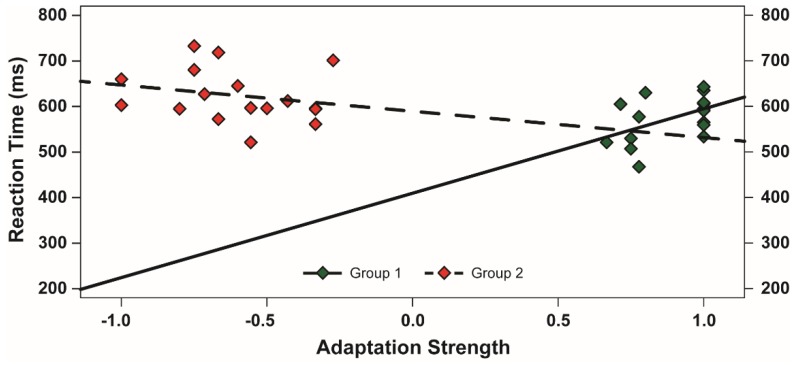
Relationship between the strength of McCollough effect induced in Phase 1 and performance in the categorization task, separately for participants in Group 1 (green diamonds and solid line) and Group 2 (red diamonds and dashed line).

A Pearson correlation analysis was conducted to attest the relationship between the strength of the McCollough effect and the categorization times, separately for each group of participants. The strength of McCollough effect was defined as the differential proportion of WV perceived as being tinged with light green and red ((WH frequencies on the green series—WH frequencies on the red series)/(WH frequencies on the green series + WH frequencies on the red series)). This value ranged between −1 and 1, where −1 indicated that WH was always perceived as being tinged with red and 1 that WV was always perceived as being tinged with green. As shown in Figure 5, there was a direct relationship between the strength of the McCollough effect and the categorization times in Group 1 (participants adapted to black and green horizontal gratings; *r*(15) = 0.40, *p* < 0.05). In contrast, no relationship was found in Group 2 (participants adapted to black and green vertical gratings; *r*(15) = −0.22, *p* = 0.39, *n.s.*).

## 4. Discussion

In this exploratory study, we investigated the conceptual processing of nouns referring to natural objects characterized by highly typical colors and orientations (e.g., pine, lizard) when the noun presentation was preceded by a selective adaptation of color-edge sensitive neurons in posterior LV4 region of the visual cortex. We found that neural adaptation exerted a robust and systematic effect on subjects’ performance. However, in contrast to our predictions, the adaptation of neurons jointly sensitive to a given color and orientation did not interfere with the conceptual processing of objects typically characterized by these features. Rather, the specific adaptation to green-and-black vertical gratings led to a general impairment of the conceptual processing of natural entities, regardless of the characteristics of the to-be-categorized nouns. Indeed, the responses of participants adapted to green-and-black vertical gratings were significantly slower than those of participants adapted to green-and-black horizontal gratings. In sum, the deactivation of neurons jointly sensitive to the green color and the vertical orientation interfered not only with the conceptual processing of green-vertical objects, but extended to all the instances of natural entities conveyed by the nouns used in the study.

One possibility is that the color-edge neurons sensitive to the green color and the vertical orientation are active whenever one has access to an instance of the category of natural entity. This possibility is further supported by the correlation analysis performed to test the relationship between the strength of adaptation induced using the McCollough procedure and the categorization times. Indeed, we found that the conceptual processing of the participants of Group 2 (who were adapted to black and green vertical gratings) was unaffected by the adaptation strength. This suggests that when neurons selectively sensitive to the green color and the vertical orientation were deactivated, they could be no longer recruited during the conceptual processing. In contrast, the conceptual processing of the participants in Group 1 (who were adapted to black and green horizontal gratings) was significantly slowed down as the adaptation strength increased, indicating that when an exaggerated activity of neurons jointly sensitive to the green color and the vertical orientation was induced by deactivating their opponent neurons (*i.e.*, neurons selectively sensitive to the red color and the vertical orientation), the former could be still used to operate the conceptual processing, but in an inverse degree to that they were busied in perceptual modality.

Why did the temporary deactivation of LV4 neurons jointly sensitive to the green color and the vertical orientation selectively impaired the access to all the nouns referring to natural entities? Admittedly, we do not have a principled explanation. One speculative explanation, that requires further studies to be better characterized and possibly confirmed, is that these perceptual features typically characterize plants. Plants are indeed, along with animals, among the most numerous members of the category of the natural entity category. In fact, plants possess these surface features in a very stable way as they contain in their tissues chlorophyll which is a poor absorber of the green portion of the spectrum, and, simultaneously, exhibit phototropism (*i.e.*, the directional growth of plants towards the sun that typically causes the plants to have vertically elongated cells). In contrast, animals generally show much more regularity in their biological motion than in their surface features which can dramatically vary even across exemplars of the same species. Given the tight and relatively stable association between plants and their surface features, the recruitment of visual neurons jointly sensitive to the green color and the vertical orientation may be extremely important, if not crucial, for accessing the general concept of natural entity since it may namely allow to reactivate the most representative and reliable perceptual experience that natural entities offer to their observers.

The results of early neuropsychological studies seem consistent with this speculative explanation. Indeed, these studies consistently reported the activation of modality-specific information during concept acquisition and retrieval (e.g., [34]). Recently, it has been suggested that fine-grained categorical knowledge may arise not only as a consequence of differing weighting values of the information from each of the major sensory/motor modalities, but also as a consequence of different weighting values of more specialized channels within each modality (e.g., [35]). For example, Crutch and Warrington [36] claimed that the categorization of animals and plants differs since the latter relies more upon sensory input including color and visual-tactile form and the former is more dependent upon input from the visual sub-channel relative to biological motion.

The relative importance of input from different circuits of sensory/motor modalities is thought to form the basis for how subordinate and basic level concepts (e.g., dog and cocker) are initially formed and then used. Our data suggest there is a possibility that differential weighted activation of modality-specific channels also constrains the access to the superordinate concept of natural entity. This explanation clearly requires further specifications about the possible mechanisms that assign weighting values to the entries from the various modality-specific channels. As far as subordinate and basic concepts are concerned, a mechanism based on channel activation rate would be sufficient: heavier weights would go to input from channels with higher frequency of activation. Behaviorally, this implies that objects that possess the same or a similar set of physical features are likely to be assigned to the same category as they constantly recruit the same modality-specific channels. However, this mechanism does not explain what happens for superordinate concepts. It has been suggested that superordinate concepts activate their subordinate members through an “instantiation principle” [37,38,39], since the physical features characterizing these concepts markedly differ. Hence, in the case of superordinate concepts the mechanism for weighting input from different specific-modality channels must rely on some sort of combinatorial principle.

Vision scientists have already faced the same problem when trying to explain how the brain combines redundant information from different modalities to form a coherent perception of external objects (e.g., [40]). It has been suggested that the optimal way for integrating noisy and potentially ambiguous multiple sources of information is to reduce the variance of this integration as much as possible by using a linear weighting rule (see [41] for other models of integration). According to the maximum likelihood estimate model [42], the most reliable sensory integration is attained by weighting of sensory signals from different modalities based on their variability. More specifically, perceptual integration results from the weighted sum of individual signals with weights proportional to the inverse of their variance. In keeping with this model, we can speculate that conceptual processing may primarily engage modality-specific channels that give rise to signals with high reliability during actual perception. Saying it differently, heavier weights would go to entries from channels with lower variability in their activation. Thus, using the concept of natural entities would entail the recruitment of neurons jointly sensitive to the green color and the vertical orientation not because from birth their activation always underlies the experience of natural entities (which is patently false), but because the variance of their activation is lower than that associated with the other modality-specific channels. This is in line with the idea that prototypes of categories are the sum of attributes having a different weight depending on a variety of factors, such as the typicality of the category members and their diagnosticity for the task at hand [43,44]. In our case the features “green” and “vertical” have a high weight because of the specific paradigm we used and because these characteristics are more stable than others across the category members.

## 5. Conclusions

In conclusion, the present study provides evidence that neural adaptation can impair conceptual processing. This evidence cannot be easily accommodated within a view according to which the dynamics of activation flow cascades from a disembodied conceptual system to the perceptual and motor systems (e.g., [7]). At the same time, these results challenge also the grounded cognition approach, at least in its simplest forms, that stipulates that whenever a semantic concept is activated the perceptual properties of that concept are activated as well [45]. More notably, our work offers novel insights into how conceptual processing makes partial use of perceptual systems suggesting that retrieving a concept entails the activation of modality-specific neural channels with weights proportional to the reliability of the signals produced by these channels during actual perception of the conceptual referents. However, this happens regardless of whether or not the referent possesses some features, provided that those features are typically associated with the corresponding superordinate category.

As Barsalou [1] noted, although the cortical areas that underlie perception and concept processing must differ in important ways, the latter “selects and stores a subset of the active neurons in a perceptual state”. Our study gives some initial hints that this selection and storing may be based on the same mechanism that mediates the integration of multiple perceptual information into a coherent percept, that is the weighting of concurrent inputs based on their reliability.

## References

[1] Barsalou L.W. (1999). Perceptual symbol systems. Behav. Brain Sci..

[2] Barsalou L.W. (2008). Grounded Cognition. Annu. Rev. Psychol..

[3] Fisher M.H., Zwaan R.A. (2008). Embodied language: A review of the role of the motor system in language comprehension. Q. J. Exp. Psychol..

[4] Glenberg A.M., Gallese V. (2012). Action-based language: A theory of language acquisition, comprehension, and production. Cortex.

[5] Jirak D., Menz M.M., Buccino G., Borghi A.M., Binkofski F. (2010). Grasping language—A short story on embodiment. Conscious.Cogn..

[6] Mahon B.Z., Caramazza A. (2005). The orchestration of the sensory-motor systems: Clues from neuropsychology. Cogn. Neuropsychol..

[7] Mahon B.Z., Caramazza A. (2008). A critical look at the embodied cognition hypothesis and a new proposal for grounding conceptual content. J. Physiol. (Paris).

[8] Martin A. (2007). The representation of object concepts in the brain. Annu. Rev. Psychol..

[9] Meteyard L., Cuadrado S.R., Bahrami B., Vigliocco G. (2012). Coming of age: A review of embodiment and the neuroscience of semantics. Cortex.

[10] Toni I., De Lange F.P., Noordzij M.L., Hagoort P. (2008). Language beyond action. J. Physiol. (Paris).

[11] Pulvermüller F., Shtyrov Y., Ilmoniemi R. (2005). Brain signatures of meaning access in action word recognition. J. Cogn. Neurosci..

[12] Marino B.F., Gough P.M., Gallese V., Riggio L., Buccino G. (2013). How the motor system handles nouns: A behavioral study. Psychol. Res..

[13] Marino B.F., Sirianni M., Volta R., Magliocco F., Silipo F., Quattrone A., Buccino G. (2014). Viewing photos and reading nouns of natural graspable objects similarly modulate motor responses. Front. Hum. Neurosci..

[14] González J., Barros-Loscertales A., Pulvermüller F., Meseguer V., Sanjuán A., Belloch V., Avila C. (2006). Reading cinnamon activates olfactory brain regions. Neuroimage.

[15] Simmons W.K., Ramjee V., Beauchamp M.S., McRae K., Martin A., Barsalou L.W. (2007). A common neural substrate for perceiving and knowing about color. Neuropsychologia.

[16] Pecher D., Zeelenberg R., Barsalou L.W. (2003). Verifying different-modality properties for concepts produces switching costs. Psychol. Sci..

[17] Chatterjee A. (2010). Disembodying cognition. Lang. Cogn..

[18] Dove G. (2011). On the need for embodied and dis-embodied cognition. Front. Psychol..

[19] Grill-Spector K., Henson R., Martin A. (2006). Repetition and the brain: Neural models of stimulus-specific effects. Trends Cogn. Sci..

[20] Morita T., Kochiyama T., Okada T., Yonekura Y., Matsumura M., Sadato N. (2004). The neural substrates of conscious color perception demonstrated using fMRI. Neuroimage.

[21] Jones P.D., Holding D.H. (1975). Extremely long-term persistence of the McCollough effect. J. Exp. Psychol.: Hum. Percept. Perform..

[22] McCollough C. (1965). Color adaptation of edge-detectors in the human visual system. Science.

[23] Barnes J., Howard R., Senior C., Brammer M., Bullmore E.T., Simmons A., David A.S. (1999). The functional anatomy of the McCollough contingent colour after-effect. Neuroreport.

[24] Kurby C.A., Wiemer-Hastings K., Forbus K., Gentner D., Regier T. (2004). Adaptation effects on world recognition times: Evidence for perceptual representations. Proceedings of the 26th Annual Conference of the Cognitive Science Society.

[25] Wiemer-Hastings K., Kurby C.A., Bara B.G., Barsalou L.W., Bucciarelli M. (2005). Access to perceptual features during world recognition. Proceedings of the 27th Annual Conference of the Cognitive Science Society.

[26] Ishihara S. (1971). Tests for Colour-Blindness.

[27] (1996). E-Prime.

[28] Laudanna A., Thorton A., Brown G., Burani C., Marconi L., Bolasco S., Lebart L., Salem A. (1995). Un corpus dell’italiano scritto contemporaneo dalla parte del ricevente. III Giornate Internazionali di Analisi Statistica dei dati Testuali.

[29] Baayen R.H., Davidson D.J., Bates D.M. (2008). Mixed-effects modeling with crossed random effects for subjects and items. J. Memory Lang..

[30] Baayen R.H., Milin P. (2010). Analyzing reaction times. Int. J. Psychol. Res..

[31] Kuznetsova A., Brockhoff P.B., Christensen R.H.B. lmerTest: Tests in Linear Mixed Effects Models. http://CRAN.R-project.org/package=lmerTest.

[32] R Core Team (2014). R: A Language and Environment for Statistical Computing.

[33] Guilford J.P., Guilford J.P. (1954). The method of pair comparisons. Psychometric Methods.

[34] Warrington E.K. (1975). The selective impairment of semantic memory. Q. J. Exp. Psychol..

[35] Warrington E.K., McCarthy R.A. (1987). Categories of knowledge. Further fractionations and an attempted integration. Brain.

[36] Crutch S.J., Warrington E.K. (2003). The selective impairment of fruit and vegetable knowledge: A multiple processing channels account of fine-grain category specificity. Cogn. Neuropsychol..

[37] Borghi A.M., Caramelli N., Setti A. (2005). Conceptual information on objects’ locations. Brain Lang..

[38] De Wilde E., Vanoverberghe V., Storms G., de Boeck P. (2003). The instantiation principle re-evaluated. Memory.

[39] Heit E., Barsalou L.W. (1996). The instantiation principle in natural categories. Memory.

[40] Stein B.E., Meredith M.A. (1993). The Merging of Senses.

[41] Massaro D.W., Friedman D. (1990). Models of integration given multiple sources of information. Psychol. Rev..

[42] Ernst M.O., Bülthoff H.H. (2004). Merging the senses into a robust percept. Trends Cogn. Sci..

[43] Tversky A. (1977). Features of similarity. Psychol. Rev..

[44] Hampton J.A. (1998). Similarity-based categorization and fuzziness of natural categories. Cognition.

[45] Goodhew S.C., Kendall W., Ferber S., Pratt J. (2014). Setting semantics: Conceptual set can determine the physical properties that capture attention. Atten. Percept. Psychophys..

